# Portrayed emotions in the movie "Forrest Gump"

**DOI:** 10.12688/f1000research.6230.1

**Published:** 2015-04-13

**Authors:** Annika Labs, Theresa Reich, Helene Schulenburg, Manuel Boennen, Gehrke Mareike, Madleen Golz, Benita Hartigs, Nico Hoffmann, Sebastian Keil, Malú Perlow, Anne Katrin Peukmann, Lea Noell Rabe, Franca-Rosa von Sobbe, Michael Hanke

**Affiliations:** 1 Psychoinformatics lab, Department of Psychology II, University of Magdeburg, Magdeburg, 39106, Germany; 2 Centre for Behavioral Brain Sciences, Magdeburg, Germany

**Keywords:** Emotional episodes, Emotional processing cues, fMRI, Audio-visual stimulus, Forrest Gump

## Abstract

Here we present a dataset with a description of portrayed emotions in the movie ”Forrest Gump”. A total of 12 observers independently annotated emotional episodes regarding their temporal location and duration. The nature of an emotion was characterized with basic attributes, such as arousal and valence, as well as explicit emotion category labels. In addition, annotations include a record of the perceptual evidence for the presence of an emotion. Two variants of the movie were annotated separately: 1) an audio-movie version of Forrest Gump that has been used as a stimulus for the acquisition of a large public functional brain imaging dataset, and 2) the original audio-visual movie. We present reliability and consistency estimates that suggest that both stimuli can be used to study visual and auditory emotion cue processing in real-life like situations. Raw annotations from all observers are publicly released in full in order to maximize their utility for a wide range of applications and possible future extensions. In addition, aggregate time series of inter-observer agreement with respect to particular attributes of portrayed emotions are provided to facilitate adoption of these data.

## Background

Recently, we have published a dataset with high-resolution functional magnetic resonance (fMRI) data of 20 participants listening to a two-hour audio-movie version of the Hollywood feature film “Forrest Gump” ^1^. Using prolonged complex naturalistic stimuli, such as this one, is one approach to study cognitive processing in situations that resemble real-life contexts more closely than controlled laboratory settings typically employed to investigate individual cognitive functions in isolation ^2^. However, multidimensional stimuli and the resulting lack of experimental control can make it harder to isolate the intervening parameters ^2^. The goal of this study was to extend the information about this movie stimulus in order to enable further analysis of the already published brain imaging data as well as to investigate the utility of this particular stimulus for future studies and additional data acquisitions. To this end, we focused on a highly relevant aspect of social cognition that is, at the same time, difficult to infer from an audio-visual stimulus by means of computer algorithms: portrayed emotion (the expression of the emotional state of a movie character by an actor).

The presentation of movies (clips) to elicit emotional responses is an established component of emotion research, in particular with respect to more differentiated emotional states, such as remorse or pride, that go beyond primary emotions, like fear, in complexity and time-scale ^3^. Emotion cues in movies can be manifold: facial expressions (e.g. smiling) ^4^, verbal cues (e.g. swearing), voice characteristics (e.g. trembling voice) ^5^, or even context cues that can be used to reason about emotional aspects of a situation based on abstract causal principles rather than direct perceptual cues ^6^. Moreover, the emotional response to a stimulus in a real-life setting is further dependent on additional factors. For example, observing a facial expression of sadness may yield an emotional response of pity or satisfaction, depending on whether the person is a friend or a punished offender of social norms. A smiling face is not an expression of happiness when the larger context identifies it as a strategy to avoid unpleasant social interactions (a fake smile). Consequently, labeling portrayed emotions in feature films is a task that requires human observers to perform complex judgments.

Two groups of frameworks for systematic description of emotions are distinguished in the literature: a) schemes with discrete emotions (labels) and b) dimensional models ^7^. Models using discrete emotion labels vary considerably in the number of differentiated emotion states. Many theories assume few basic, innate emotions ^8^; others discriminate up to 36 affective categories ^9^. Dimensional models, for example the circumplex model, locate different emotional states in a two-dimensional space, commonly using the axes arousal and valence ^10^.

Current emotion assessment tools, like the Facial Action Coding System (FACS) or the Specific Affect Coding System (SPAFF) ^11^, attempt to combine both approaches. However, they typically involve following complex instructions regarding the interpretation of facial expressions or other physical and cultural indicators of emotion ( 11, p. 281) and thus require an intensive training of observers. While these tools provide reliable procedures for rating emotions, they are not very intuitive and are consequently only accessible to experts.

In this study, the primary goal was not to generate an objective labeling of portrayed emotions in the Forrest Gump movie. Congruent with our goal to enable further studies of already published brain imaging data, we rather aimed at producing a description of the emotional content of the Forrest Gump movie stimulus as it was likely perceived by the participants in our past and future brain imaging studies (potentially biased by age, native language, or education). Therefore, our approach was to collect data from multiple observers that stem from the same student population, using a simplified procedure that does not require extensive training.

Portrayed emotions were independently annotated for the audio-only version of “Forrest Gump” (used in Hanke M *et al.* ^1^) and for the original audio-visual movie in order to obtain information about which aspects of portrayed emotions are congruent between both stimulus variants.

The resulting dataset of annotations of portrayed emotions combines a dimensional rating with a categorical labeling of emotions and a description of their associated perceptual evidence. In the following, we provide evidence that our procedure yielded a reliable description that can be used to segment the movie into episodes of portrayed emotions. Moreover, we show that the time course of inter-observer agreement (IOA) with respect to many aspects of portrayed emotion is a reliable measure of their perceptual ambiguity throughout the movie.

### Possible applications

In combination with the already publicly available brain imaging data, these annotations form a two-hour high resolution fMRI measurement for auditory emotion cue processing from 20 participants. With the addition of a future publication of brain imaging data recorded from a stimulation with the audio-visual movie, the full dataset will enable comparative studies investigating the processing of rich emotional stimuli via different sensory pathways. Moreover, these new annotations of portrayed emotions are another step towards a comprehensive description of this reproducible movie stimulus ^1^ and improve its general utility for independent studies on social cognition with a focus on the perception of emotions in real-life situations.

## Materials and methods

### Stimulus

The annotated stimulus was a slightly shortened version of the movie “Forrest Gump” (R. Zemeckis, Paramount Pictures, 1994). Two different variants of this movie were annotated separately. The first was a German audio-description as broadcast as an additional audio track for visually impaired listeners on Swiss public television (Koop, Michalski, Beckmann, Meinhardt & Benecke produced by Bayrischer Rundfunk, 2009). This audio-only stimulus was the same as the one used in Hanke M *et al.* ^1^, and is largely identical to the dubbed German soundtrack of the movie except for interspersed narrations by a male speaker who describes the visual content of a scene. These descriptions take place when there is no dialog, off-screen speech, or other relevant audio-content in the movie. The second variant is the audio-visual movie with the original dubbed German soundtrack (without additional narrations) and the exact same timing (and cut scenes) as the audio-only version ( 1, contains instructions on how to reproduce the stimulus from the DVD release).

### Observers

Annotations were created by the authors themselves as part of a practical course on scientific observation. A total of 12 students at the Otto-von-Guericke-University in Magdeburg, Germany participated in this effort and received course credit. Based on personal preference, students assigned themselves to one of two groups tasked with the annotation of portrayed emotion in either the audio-visual movie, or the audio-only version. The audiovisual group consisted of 9 students (all female), the audio-only group comprised 3 students (all male). This gender bias could have had an impact on the perceived emotions. None of the observers participated in the previous brain imaging study ^1^.

### Procedure

Annotations were performed with the help of Advene ^12^, a dedicated open-source video annotation tool that offers convenient navigation of structural units of the stimulus and provided uniform position information with subsecond precision. Each observer annotated all of 205 previously identified movie scenes in an individual random order to minimize “carry-over” effects and help observers focus on current indicators of portrayed emotions in contrast to more slowly changing characteristics, such as mood, or other biases introduced by the movie plot. Scenes were used as units for annotation, because transitions between them typically involve a change of location or fast progression of time that make persistence of the nature of portrayed emotions across scenes less likely. Observers used a scene annotation of the movie imported into Advene to aid navigation within the movie. They only had to click on the visual representation of a scene (a numbered box) in a time line display to start the playback at the start of a scene. Advene could also be configured to play a selected scene in a continuous loop. For composing the annotation content, observers used a separate spreadsheet application and not the data input features of Advene.

Observers were instructed to work alone in a setting free of distractions and to use headphones for optimal saliency of the stimulus. There was no strict guideline regarding the length of an annotation session, but observers were informed to stop working for any number of breaks whenever they could no longer guarantee a high level of attention for the task. Sessions were typically spread over multiple days over the course of three weeks. On average, observers reported that it required around 30 hours each to complete the annotation.

### Annotation content

Each observer collected annotations in a spreadsheet with columns for start time and end time of an emotion; a label of the movie character portraying the emotion; variables for valence, arousal, and direction of the emotion as well as an optional emotion category label; and a list of identified indicators for the start and end of an emotion. Each of these variables are described in more detail in the remainder of this section. In addition, observers also recorded the index of the scene containing a respective emotion. This variable was only used for error detection (mismatch of emotion duration with the start and end time of a scene) and is not included in the published dataset.

**Start and end time**  The start time identifies the onset of the first observed indicator of an emotion. Likewise, the end time corresponds to the offset of an emotion — defined as the time where no evidence for an emotion is present anymore. Observers were instructed to aggregate short segments of uniform emotional episodes into a single annotation. For example, if a character was happy and smiling throughout a long period in a scene, the episode would span all the time from the beginning to the end of the smile even if the character’s face was not visible the entire period, as long as no evidence of a change of emotion was found. All times are reported in seconds. Although Advene offers time stamps with the precision of the movie frame rate (25 frames per second), pre-tests revealed that the temporal accuracy of human observers for complex emotion indicators, like a developing facial expression, is much coarser. In order to reduce the chance of unrecoverable typos in the time stamps, observers were instructed to record timing information with second-precision only.

**Onset and offset cues**  Onset indicators describe what kind of evidence for an emotion was detected in the movie stimulus. We distinguished facial expressions, gestures or body language, context information, verbal, and non-verbal audio cues. Observers could record multiple onset indicators. Despite the term “onset”, these indicators did not all have to be present at the very beginning of an emotional episode. For example, an extended period of sadness could start with a facial expression and later on add a congruent body language cue. In that case, the respective labels were aggregated into a list. In the audio-only stimulus, the narrator was frequently the only source of information regarding the emotional state of a character; thus, we included a dedicated category for this scenario.

In addition to onset indicators, observers also recorded the type of evidence for the end of an emotional episode. Four conditions were distinguished: 1) changing from one emotion to another 2) entering a neutral emotional state 3) a character leaving an ongoing scene with no further evidence for its emotional state 4) the end of a scene. Table 1 describes all distinguished cue categories.

**Table 1.** Categories for cues indicating the onset or offset of an emotion.

Label	Description
*Onset* AUDIO CONTEXT GESTURE FACE NARRATOR VERBAL	Non-verbal audio cues, feelings expressed without words. For example: laughing, crying, wheezing, sighing Emotion portrayed through context information and not directly by a character. For example, a social rejection of an individual by a group without visible emotional response Body language cues Facial expressions Description of an emotion given by the narrator (only for audio stimulus) Emotion expressed through words either as a direct description (e.g., a self-report) or by means of a particular choice of words (e.g., harsh language)
*Offset* EMOCHANGE EMOFADED CHARLEFT SCENEEND	Present emotion is replaced by another, different emotion Emotion fades with a change to an emotion neutral state Character leaves a scene (no further evidence for an emotion) Scene transition with a change of time or location indicates the end of an emotion

**Character label**  This label identifies which movie character is portraying an emotion. Observers were provided with a list of character labels that was derived from an annotation of the movie’s dialog in order to achieve a consistent labeling. However, some characters portray emotions but have no lines of dialog, and, in other instances, observers chose inconsistent labels. During data curation, character labels were therefore manually consolidated into a set of 35 categories that uniformize labeling across observers. The full list of character labels is shown in table 2 and was derived using the following rules: a) main characters and famous/familiar characters receive individual labels as do the two narrator voices b) relatives of main characters are labeled according to their relation c) all other characters are consolidated into generic categories that preserve gender, age (children, adults, or seniors), and number (individual or group).

**Table 2.** Consolidated character labels. Any annotation of a portrayed emotion is associated with exactly one of these 36 labels. While main characters, their relatives, and famous characters have individual labels, all remaining characters in the movie are aggregated into generic categories that preserve information on gender, age group, and number.

Character label	Description	Character label	Description
*Main characters* **BUBBA** **DAN** **FORREST**	Forrest’s army pal Lt. Dan adult Forrest Gump	**FORRESTJR** **JENNY**	young Forrest Gump Forrest’s love
*Special* **FORRESTVO**	voice-over narrator Forrest	**NARRATOR**	audio-description narrator
*Famous characters* **ABBIEHOFFMAN** **BOBHOPE** **DICKCAVETT** **ELVISPRESLEY** **JOHNLENNON**	activist (lookalike actor) entertainer (orig. footage) talk master (orig. footage) The King (orig. footage and lookalike actor) Beatle (orig. footage)	**NEILARMSTRONG** **PRESIDENTJOHNSON** **PRESIDENTKENNEDY** **PRESIDENTNIXON** **ROBERTKENNEDY**	first man on the moon (orig. footage) US-president (orig. footage) US-president (orig. footage) US-president (orig. footage) Brother of J. F. Kennedey (orig. footage)
*Relatives of main characters* **BUBBASGRGRANDMA** **BUBBASGRGRGRANDMA** **BUBBASYOUNGBROTHER** **JENNYSDAD**	Bubba’s great-grandmother Bubba’s great-great-grand-mother Bubba’s young brother Jenny’s father	**JENNYSGRANDMA** **MRSBLUE** **MRSGUMP**	Jenny’s grandmother Bubbas’s mother Forrest’s mother
*Generic character categories* **CHILDREN** **GIRL** **BOY** **OLDERBOYS** **MAN** **OLDMAN**	group of children individual girl individual boy group of older boys individual man individual old man	**MEN** **OLDMEN** **WOMAN** **OLDWOMAN** **WOMEN** **CROWD**	group of men group of old men individual woman old woman group of women group of people with mixed gender

It is important to note that only emotions portrayed by one or more movie characters were recorded. This excludes other potentially emotion-inducing stimuli (e.g, music in the soundtrack) or inanimate stimuli (e.g., the depiction of a beautiful sunset or chirping birds).

**Valence, arousal, and direction**  Binary variables were used to record the valence of an emotion (positive vs. negative), the state of arousal of the character portraying it (high vs. low), and whether the emotion is directed towards the character itself or another one (e.g. feeling pity for somebody). Together, these three indicator variables offer a coarse categorization of observed emotions in the spirit of dimensional models of emotion.

Arousal was considered a global indicator of the emotional status of a character, i.e., for any given time in the movie annotations, indicate either a high or low level of arousal for a character — but never both at the same time. Unlike arousal, valence and direction indicators were considered non-exclusive, i.e., a character could show signs of both positive and negative emotions at the same time. Likewise, a character could simultaneously exhibit self-directed emotions and emotions directed towards others. In order to annotate such situations, observers had to create records for two or more portrayed emotions with overlapping timing.

**Emotion labels**  Observers were instructed to assign one of 22 labels for discrete emotion categories. These categories were derived from a schema proposed by Ortony ^13^ and concrete definitions were developed based on the Specific Affect Coding System ^11^. The complete list of emotions is shown in table 3. Pre-tests using a larger and more detailed set of emotion categories revealed weak agreement between observers for a forced categorization of emotional episodes. Based on these results, we decided to make this part of the annotation optional, and observers were instructed to assign an emotion label only if they saw a “perfect” match with any of the categories.

**Table 3.** List of 22 emotion categories the observer could (optionally) use to further qualify the nature of a portrayed emotion.

Label	Description
ADMIRATION ANGERRAGE COMPASSION CONTEMPT DISAPPOINTMENT FEAR FEARCONFIRMED GLOATING GRATIFICATION GRATITUDE HAPPINESS HAPPYFOR HATE HOPE LOVE PRIDE RELIEF REMORSE RESENTMENT SADNESS SATISFACTION SHAME	Appreciation of another person or object Response to injured respect or autonomy of a person by another one Concern for someone’s suffering Disdain of another person or object When expectations were not met Triggered by a dangerous situation The confirmation of the prospect of an undesirable event To be happy because another person suffers Triggered by a positive event for which one credits themselves Triggered by a positive event for which one credits another person Pleasant and joyful condition concern in happiness of someone else Rejection of another person, intense dislike, or animosity Positive expectation for a person or special event in the future Affection for another person Positive reflection of accomplishments and status A worry/negative expectation that does not come to pass Triggered by a negative event initiated by themselves Response to perceived unfair treatment Emotional pain after loss or misfortune When expectations get fulfilled Dissatisfaction with one’s own behavior, because norms are violated

### Quality control

We used an automated procedure to check annotation records of individual observer for errors or potential problems. Observers submitted their annotations in tabular form to a script that generated a list of error and warning messages. Using this feedback, observers double-checked their annotations as often as necessary until no objective errors were found and all warning messages were confirmed to be false positives. The following annotation properties were tested:

- start time had to precede end time (error)
- no character label (error)
- no onset cue label (error)
- missing arousal, valence, or direction value (error)
- conflicting simultaneous arousal annotation for the same character (error)
- emotion portrayed for longer than a minute (warning)
- annotation starts prior to a scene start or ends after a scene end (warning)
- unrecognized code/label for any variable (warning)
- no offset cue label (warning)

### Dataset content

The released data comprises four components: 1) raw annotations 2) aggregate inter-observer agreement (IOA) time series 3) emotion episode segmentations 4) the source code to generate all data derived from the raw annotations and all summary statistics presented in the Data Note.

**Raw annotations**  In order to maximize the utility of the annotations, they are released in full rather than only as aggregate information. The annotations from each observer are available in an individual plain text file with comma-separated value (CSV) markup in the raw directory. This file format facilitates import and further processing in virtually any software application that supports data in tabular form. The file names indicate the annotated stimulus type (prefix av for the audio-visual movie and ao for the audio-only movie) as well as the observer identity.

Each file contains nine columns that correspond to the annotation properties described above. start, end) start and end time of an emotion episode reported as seconds from the start of the movie; character) a single character label from the set shown in table 2 indicating the character portraying the emotion; arousal) state of arousal indicated with the labels LOW or HIGH; valence) emotional valence indicated with the label POS or NEG; direction) direction of the emotion indicated with the labels SELF or OTHER; emotion) a space-separated list of emotion labels from the set shown in table 3; oncue) a space-separated list of onset indicators for a portrayed emotion from the set shown in table 1; and offcue) a space-separated list of offset indicators from the set also shown in table 1. With the exception of the columns emotion and offcue, all variables are considered mandatory and are present for all annotations. The remaining columns contain optional attributes and can be empty.

**Inter-observer agreement (IOA) time series**  IOA time series reflect the consistency of observations at any particular time in the movie. The time series amplitude corresponds to the fraction of observers indicating the presence of a particular attribute of an emotion episode (interval [0, 1]). Time series for the three bipolar attributes *arousal*, *valence*, and *direction* were computed by subtracting the IOA with respect to the presence of the both extremes from each other. For example, the time series for *arousal* was computed by subtracting the time series of the IOA for low-arousal episodes from the time series of high-arousal episodes. This results in values from an interval of [−1, 1] — where −1 corresponds to perfect agreement with respect to the presence of a low-arousal episode and +1 indicates perfect agreement regarding high arousal.

IOA time series were computed for three different segment durations (i.e., agreement is considered when observers indicate the presence of a particular attribute anywhere in a segment): 1 s (temporal precision of the annotations), 2 s (corresponding sampling rate of the available brain imaging data), and the actual location and duration of individual shots in the movie (median duration ≈5 s). Individual time series for the three bipolar attributes, all 22 emotion categories, and the six emotion onset cues are available in the timeseries directory. The file names are encoded as follows: prefix ioats, followed by a label for the segment duration (e.g. 1s), followed by the stimulus label ( av or ao), and a character label (e.g. forrest, see table 2) or allchar for an aggregate time series across all characters. All files are in plain text format with CSV markup. The column headers indicate the corresponding emotion attribute.

**Emotion episode segmentation**  Based on IOA time series, a segmentation of the movie into episodes of portrayed emotion was performed for each of the 35 character categories individually. IOA time series for 33 attributes (arousal (bipolar), valence and direction (separate for each state), 22 emotion category labels, and six onset cues) were binarized by thresholding with a minimum absolute IOA of 50%. An episode of portrayed emotion was defined as a time window with at least one super-threshold emotion attribute. The duration of an episode was determined by the number of consecutive movie segment without a change in the unique combination of super threshold attributes.

Emotion episodes for all characters are available in the segmentation directory in plain-text files with tab-separated value (TSV) markup. The file format follows the conventions required by the Advene software for “simple-structured content”, but are also compatible with generic spreadsheet applications. Each file contains three columns (without a header). The two columns contain the start and end time of an episode in seconds. The third column contains a space-separated list of key/value pairs. Five such properties are present for each episode: char) the label of the character portraying the emotion; tags) a comma-separated list of tags corresponding to all super-threshold emotion attributes; arousal, val_pos, and val_neg) these three properties each correspond to the median IOA value across the entire episode for arousal (bipolar) and positive and negative valence (each unipolar). The names of all tags typically correspond to the lower-case name of the attribute, with the exception of the three bipolar attributes where each extreme is coded with a dedicated label (arousal: ha/la (high/low), valence: pos/neg, direction: self/other).

Emotion episode segmentation was performed separately for the audio-visual and the audio-only movie—once using IOA time series sampled with a 1 s segment size and a second time sampled with the actual duration and location of shots in the audio-visual movie. File names encode these conditions as follows: prefix emotions followed by the stimulus label ( av or ao) followed by the segment size label ( 1s or shots).

**Source code**  The full source code for all descriptive statistics and figures included in this paper is available in descr_stats.py (Python script). Moreover, this script also contains all functionality to generate the IOA time series as well as the emotion episode segmentation from the raw annotations in the data release. The required information on the timing of movie scenes and shots is contained in two additional CSV files: movie_scenes.csv and movie_shots.csv.

### Time stamp conversion

To be able to use the provided emotion annotations for an analysis of functional data (fMRI, cardiac trace, etc.) acquired using the previously described procedure ^1^, timing information has to be converted because data were recorded in eight sessions using partially overlapping segments of the movie. Table 4 specifies the location of these segments with respect to the timing of the unsegmented movie used for emotion annotation. Subtracting movie segment start times from annotation time stamps yields valid relative time stamps with respect to the time series of the available functional data.

**Table 4.** Position of the fMRI stimulus segments with respect to the movie annotation time. The last column lists the position of the scene boundary used as a reference for the segment transition. All times are in seconds and refer to the full (unsegmented) stimulus, which is a shorted version of the original movie ^1^.

#	Start	End	Duration	Boundary
0 1 2 3 4 5 6 7	0.0 886.0 1752.0 2612.0 3572.0 4480.0 5342.0 6410.0	902.0 1768.0 2628.0 3588.0 4496.0 5358.0 6426.0 7086.0	902.0 882.0 876.0 976.0 924.0 878.0 1084.0 676.0	891.2 1759.2 2618.8 3578.5 4488.0 5349.2 6418.2 7085.5

## Dataset validation

**Figure 1.**
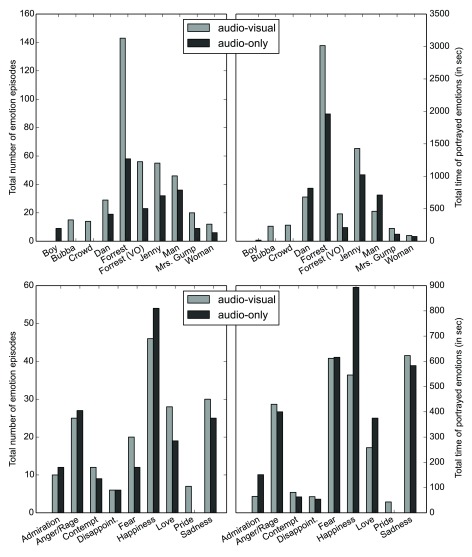
Annotation frequencies by character and emotion. The minimum criterion for counting emotion episodes is a 50% inter-observer agreement. Only categories/characters with a minimum of five such episodes are shown. The left panels show the number of episodes for both movie variants; the right panels show the cumulative duration of emotion display across all considered episodes. The top panels show the annotation frequency by movie character; the bottom panels show the corresponding frequencies for emotion categories.

**Characters portraying emotions**  Annotations for a total of 34 and 27 different character categories were created for the audio-visual movie (AV) and the audio-only movie (AO) respectively. In the AV case, these comprise all possible characters except the narrator of the audio-description. There are 9 (AV) and 8 (AO) characters for which there is a 50% minimum inter-rater agreement on the presence of a portrayed emotion for at least five episodes of one second or longer throughout the entire movie (arbitrary thresholds). The number of emotion episodes for the AV/AO union of these characters sets is shown in figure 1 (top). As expected, the majority of all portrayed emotions are associated with the main movie characters Forrest, Dan, and Jenny.

**Portrayed emotion categories**  For the audio movie, 97.5% of all annotations include at least one of the 22 explicit emotion labels (see table 3), whereas only 68.4% of annotations for the audio-visual movie contain such a label. This is an indication that emotion cues in the visual movie are more diverse and ambiguous in comparison to the audio-only stimulus. Figure 1 (bottom) shows the frequency and display duration for individual emotions. For both stimulus types, the majority of all emotional episodes involve the five categories anger/rage, fear, happiness, love, and sadness. Some of our pre-determined emotion categories were not attributed to any emotion episodes in the movie, were used very infrequently, or were used only by a small subset of observers. Such emotions include resentment, gratification, fears confirmed, or satisfaction (see table 5). It remains unknown whether these emotions are not portrayed in the movie or if the respective categories were not appropriately defined.

**Table 5.** Intra-stimulus inter-observer consistency. All values are Spearman correlations for 1 Hz modulations of the fraction of IOA (as, for example, depicted in figure 3) with respect to a particular emotion attribute across the entire duration of the movie. The specified range corresponds to the width of the 95% confidence interval of the mean correlation for all possible combinations of partitioning observers into two sub-groups (audio-visual 4 vs. 5; audio-only 1 vs. 2). Higher correlations indicate higher consistency of agreement modulations across observer sub-groups. Correlations higher than 0.5 (arbitrary threshold) are depicted in bold for visualization purposes. The *all characters* column indicates the agreement for a particular emotion attribute over time irrespective of the annotated character. The columns on the right show the corresponding correlations for the two main characters. *n/a* fields indicate an insufficient number of annotations to compute the consistency measure.

	All characters		Forrest-only		Jenny-only	
	Audio-visual	Audio-only	Audio-visual	Audio-only	Audio-visual	Audio-only
*Dimensional emotion attributes* Arousal Valence Direction	**0.676** ±0.006 **0.851** ±0.004 **0.555** ±0.011	0.305 ±0.078 **0.564** ±0.044 0.068 ±0.050	**0.600** ±0.008 **0.784** ±0.006 **0.543** ±0.012	0.443 ±0.078 0.440 ±0.054 -0.059 ±0.072	**0.688** ±0.006 **0.858** ±0.003 **0.576** ±0.012	0.330 ±0.076 **0.598** ±0.079 0.053 ±0.145
*Emotion categories* Admiration Anger/Rage Compassion Contempt Disappoint. Fear Fears conf. Gloating Gratification Gratitude Happiness Happy-for Hate Hope Love Pride Relief Remorse Resentment Sadness Satisfaction Shame	0.392 ±0.006 **0.719** ±0.005 0.337 ±0.010 0.433 ±0.008 0.408 ±0.008 **0.749** ±0.003 0.048 ±0.009 **0.644** ±0.014 0.155 ±0.010 0.317 ±0.012 **0.618** ±0.006 0.478 ±0.013 0.441 ±0.011 0.333 ±0.010 **0.568** ±0.008 0.473 ±0.011 0.353 ±0.019 **0.557** ±0.022 n/a **0.737** ±0.005 0.112 ±0.007 0.316 ±0.006	0.343 ±0.094 0.377 ±0.042 0.182 ±0.013 0.157 ±0.043 0.367 ±0.095 **0.546** ±0.108 n/a 0.045 ±0.038 n/a n/a 0.289 ±0.107 n/a 0.097 ±0.070 0.217 ±0.060 0.396 ±0.032 0.156 ±0.054 -0.003 ±0.001 0.323 ±0.107 n/a 0.464 ±0.023 n/a 0.185 ±0.112	0.306 ±0.015 **0.693** ±0.006 0.232 ±0.019 n/a 0.431 ±0.007 **0.729** ±0.004 n/a n/a 0.242 ±0.017 0.267 ±0.024 **0.561** ±0.007 0.408 ±0.028 0.478 ±0.027 0.202 ±0.008 0.472 ±0.008 **0.536** ±0.009 **0.505** ±0.028 n/a n/a **0.750** ±0.005 n/a 0.290 ±0.006	n/a 0.141 ±0.100 n/a n/a 0.456 ±0.129 **0.504** ±0.072 n/a n/a n/a n/a 0.381 ±0.060 n/a n/a n/a 0.327 ±0.092 n/a n/a 0.453 ±0.364 n/a **0.522** ±0.006 n/a 0.034 ±0.036	n/a **0.809** ±0.007 0.459 ±0.018 0.404 ±0.020 n/a **0.633** ±0.005 n/a n/a n/a 0.165 ±0.015 **0.738** ±0.005 n/a n/a 0.439 ±0.026 **0.660** ±0.009 n/a n/a 0.464 ±0.006 n/a **0.695** ±0.009 n/a 0.201 ±0.015	**0.643** ±0.197 **0.626** ±0.109 0.363 ±0.042 n/a n/a 0.392 ±0.013 n/a n/a n/a n/a **0.532** ±0.108 n/a n/a **0.804** ±0.052 0.432 ±0.040 n/a n/a n/a n/a 0.228 ±0.040 n/a n/a
*Emotion onset cues* Audio Context Face Gesture Narrator Verbal	0.418 ±0.008 0.384 ±0.010 **0.713** ±0.006 **0.619** ±0.010 n/a **0.637** ±0.008	0.037 ±0.051 n/a n/a n/a 0.251 ±0.047 0.368 ±0.107	0.354 ±0.013 0.424 ±0.011 **0.687** ±0.010 **0.532** ±0.011 n/a **0.565** ±0.007	n/a n/a n/a n/a 0.202 ±0.045 0.455 ±0.042	0.203 ±0.007 **0.547** ±0.010 **0.880** ±0.004 **0.769** ±0.009 n/a **0.783** ±0.005	n/a n/a n/a n/a **0.578** ±0.286 **0.626** ±0.168

**Figure 2.**
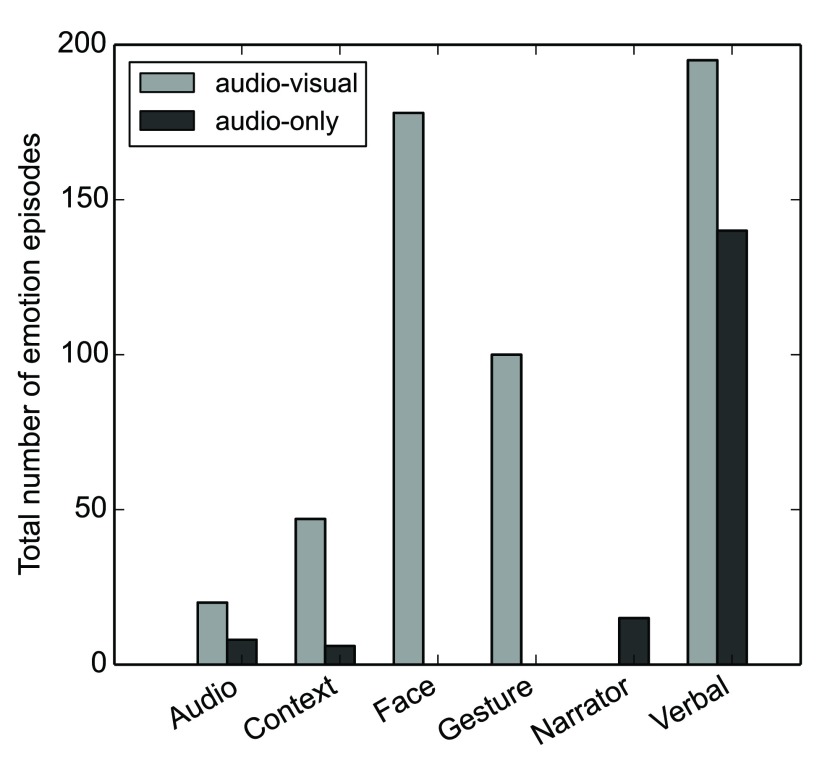
Number of emotion episodes for all distinguished onset cue categories (minimum 50% inter-rater agreement) for both stimulus types.

**Figure 3.**
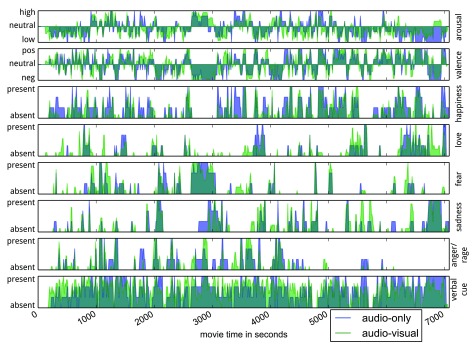
Inter-observer agreement (IOA) time courses for selected emotion attributes across the full duration of the movie. Extreme values indicate perfect agreement with respect to presence or absence of a particular emotion attribute at a given time. IOA time series for the two movie types are overlaid on top of each other. For the purpose of visualization, the depicted time series reflects IOA computed using a 10 s segment size — in contrast to 1 s used for all other statistics presented in this paper.

**Emotion onset cues** Figure 2 shows the frequency of individual emotion onset cues for all six distinguished cue types. When only considering annotations with a minimum inter-rater agreement of 50% with respect to the type of onset cue, the two dominating emotion cues are facial expressions (AV only) and verbal cues (for both stimulus types), followed by gestures and body language (AV only). Figure 3 (bottom row) shows a rather uniform distribution of verbal emotion cues across the entire duration of the movie for both stimulus types. This suggests that a comparative analysis of verbal emotion cue processing during AV vs. AO stimulation, as suggested above, may be feasible.

Annotations of portrayed emotions in the movie "Forrest Gump" See main body of text for detailed description of dataset.

### Observer agreement as a probabilistic indicator

It can be assumed that observers in this study have used individual intensity thresholds for determining the start, end, or quality of an emotional episode. Therefore, the IOA with respect to a particular aspect of portrayed emotions at any given time in the movie could be seen as a measure of the relative probability of perceiving an emotion feature. In order to assess the reliability of such a measure, we generated time series for the IOA with a sampling rate of 1 Hz, as described above, and evaluated their reliability and validity in terms of their correlation across observers, stimulus types, and with each other.

**Intra-stimulus inter-observer consistency**  The reliability of IOA time courses was estimated by repeatedly splitting the set of observers into two groups of approximately equal size. Within each group, IOA time courses where computed and correlated across the observer subgroups. Split-half correlations were computed for all possible ways of combining observers into two groups (without replacement). Table 5 shows the average time series correlation across all splits and the width of the 95% confidence interval of the mean.

In general, IOA reliability is larger for the AV movie annotations than for the AO movie. This finding is likely a result of the lower number of observers for the latter. Intra-stimulus IOA reliability estimates suggest that a subset of emotion attributes or categories is not contained in the AO movie stimulus. The most prominent example is the direction of an emotion. Emotion categories like hate, shame, or relief also show large reliability differences between stimulus types. However, in these cases the small number of emotion episodes may multiply the negative effect of the small number of observers on the interpretability of this estimate.

Uniformly high reliability estimates were found for the two main dimensional emotion attributes arousal and valence, as well as for the five most frequently annotated emotion categories anger/rage, fear, happiness, love, and sadness.

**Figure 4.**
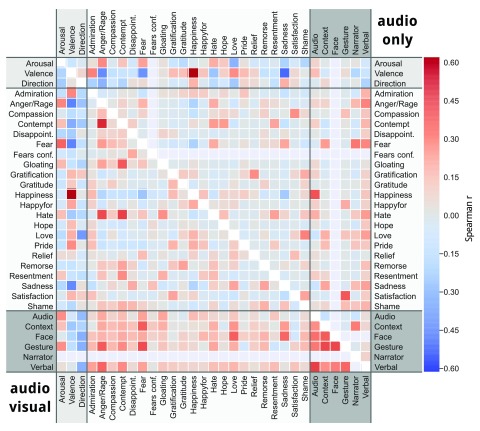
Intra-stimulus indicator correlation. The lower triangular matrix depicts the correlations between the IOA time courses for the three primary bipolar emotion attributes (arousal [high/low], valence [pos/neg], and direction [self/other]), the 22 emotion categories, and the six emotion onset indicators for the audio-visual movie. The upper triangular matrix shows the corresponding correlations for the audio-only movie. There were no observations for the emotion “fears confirmed”, or the facial expression onset cue in the audio-only movie. Likewise, there were no observations for the “narrator” onset cue in the audio-visual movie. The corresponding undefined correlations are depicted as zeros.

**Intra-stimulus inter-variable correlations**  In order to assess possible confounds in the annotations, we computed the correlation of intra-stimulus IOA time courses (across all observers) for all pairwise combinations of emotion attributes and categories. The correlation matrices for the AV and the AO movie stimulus are depicted in figure 4. The results reveal an expected correlation pattern between individual emotion categories and the two main dimensional attributes arousal and valence — with stronger correlations being observed for more primary and more frequently portrayed emotions. Interestingly, audio and verbal cues in the AV stimulus seems to be more associated with a high state of arousal and emotions directed towards other people than in the AO stimulus. In the AV stimulus, facial expressions seem to have a tendency to communicate negative emotions rather than positive ones.

**Inter-stimulus consistency**  To estimate the similarity of the two stimulus types, we computed the correlation of IOA time courses for the AV and the AO movie with respective to emotion attributes and categories. Table 6 shows the respective correlations and their 95% confidence intervals. Again, the three dimensional attributes as well as the five most frequent emotion categories show significant correlations. Notably, verbal cues are the most consistent indicator of emotions across both types of stimuli. This is evidence for an expected substantial semantic similarity with respect to the emotional content in the two variants of the “Forrest Gump” movie.

**Table 6.** Inter-stimulus indicator correlation. All values are Spearman correlations of 1 Hz modulations of the fraction of IOA (as, for example, depicted in figure 3) for the two stimulus types (audio-visual and audio-only movie). The specified range corresponds to the width of the 95% confidence interval of the correlation (computed via Fisher transformation). Correlations higher than 0.5 (arbitrary threshold) are depicted in bold for visualization purposes. *n/a* fields indicate an insufficient number of annotations to compute the consistency measure.

	All characters	Forrest-only	Jenny-only
*Dimensional emotion attributes* Arousal Valence Direction	0.474 ±0.018 **0.726** ±0.011 0.425 ±0.019	0.278 ±0.021 **0.557** ±0.016 0.295 ±0.021	**0.518** ±0.017 **0.693** ±0.012 **0.509** ±0.017
*Emotion categories* Admiration Anger/Rage Compassion Contempt Disappointment Fear Gloating Gratification Gratitude Happiness Happy-for Hate Hope Love Pride Relief Remorse Resentment Sadness Satisfaction Shame	0.260 ±0.022 0.485 ±0.018 0.232 ±0.022 0.201 ±0.022 0.372 ±0.020 **0.673** ±0.013 0.231 ±0.022 0.062 ±0.023 0.201 ±0.022 **0.532** ±0.016 0.289 ±0.021 0.266 ±0.022 0.181 ±0.022 **0.618** ±0.014 0.203 ±0.022 0.062 ±0.023 0.376 ±0.020 **0.585** ±0.015 **0.545** ±0.016 0.038 ±0.023 0.254 ±0.022	0.149 ±0.023 0.307 ±0.021 -0.018 ±0.023 n/a 0.409 ±0.019 **0.525** ±0.017 n/a -0.004 ±0.023 0.071 ±0.023 0.499 ±0.017 0.118 ±0.023 0.279 ±0.021 0.123 ±0.023 0.444 ±0.019 0.194 ±0.022 -0.007 ±0.023 **0.578** ±0.015 -0.001 ±0.023 **0.537** ±0.016 -0.007 ±0.023 0.401 ±0.019	0.197 ±0.022 0.478 ±0.018 0.357 ±0.020 0.041 ±0.023 n/a **0.799** ±0.008 n/a -0.003 ±0.023 0.444 ±0.019 **0.645** ±0.013 0.064 ±0.023 -0.001 ±0.023 0.464 ±0.018 **0.592** ±0.015 n/a n/a 0.316 ±0.021 n/a **0.653** ±0.013 n/a 0.104 ±0.023
*Emotion onset cues* Audio Context Verbal	0.236 ±0.022 0.267 ±0.021 **0.582** ±0.015	0.319 ±0.021 0.322 ±0.021 **0.560** ±0.016	0.245 ±0.022 0.316 ±0.021 **0.839** ±0.007

## Data availability

F1000Research: Dataset 1. Annotations of portrayed emotions in the movie “Forrest Gump”, 10.5256/f1000research.6230.d45328 ^14^
