# Supplementary material for: Portrayed emotions in the movie "Forrest Gump"
Source: F1000Res. 2015 Apr 13;4:92. [Version 1] doi: 10.12688/f1000research.6230.1 (PMC4416536; doi:10.12688/f1000research.6230.1)
Supplement: Peer review history

# Peer review: Referee response for version 1

**DOI:** 10.5256/f1000research.6684.r8301

**Published:** 2015-04-27

**Authors:** Ariel Rokem

**Affiliations:** 1 Department of Psychology, Stanford University, Stanford, CA, USA

This paper provides a description and analysis of a publicly released data-set: an annotation of the emotions expressed by characters in different variants of the movie Forrest Gump, in either audio or audio-visual formats. The data-set is interesting in its own right, and will be eminently useful in conjunction with the fMRI data that this group has previously released, and will continue to release. This data paper goes well beyond a standard description of the data-set, by providing analysis and interpretation of the data.

I have just one comment with regards to providing direction for eventual interpretation of the data by users: The conflation of gender differences in perceiving emotions with audio vs. visual distinctions is a real concern, and the authors acknowledge this, but the current description does not provide enough direction on how to deal with this potential confound. I think that it would be helpful if a few sentences about gender differences in perceiving emotion would be added, and some evaluation of what the effects of these differences might be in this data. For example, do differences between AO and AV annotations coincide with predictions from the gender difference literature?

It is commendable (and still sadly unusual) that that authors make software that is so easy to use, which reproduces pretty much every single statement they make about the data and produces all the figures. That said, I do have a few comments for improvements of this software:

1. Though not strictly necessary, in this day and age, it would be nice to make the scripts provided compatible with python 3 (from a quick look, it looks like the addition of some parentheses should do the trick, and see more below).
2. Making the code PEP8 compatible (e.g. line-wrapping) would make it easier to read.
3. The authors might disagree, but pymvpa seems like an onerous dependency just to supply some utility functions used in the analysis. I would add `plot_bars`, `xunique_combinations` and `unique_combinations` to the code distributed with the data. Here's a gist of the file I used when my computer refused to compile libsvm: https://gist.github.com/arokem/cd4270a78cbad58c2cd4
4. As a conclusion from the previous point, a (github) repository with the code used here would be good, for future users of this data/code to be able to make their proposals for improvements to the code via pull requests. The state of the code for this publication could be a tag/release in this repo.

Minor:

Table 3, the description of HAPPYFOR is: 1) not clear and 2) not capitalized (in contrast to the other descriptions). I am guessing you meant something like: "Like HAPPINESS, but as reflected off another character", or some-such.

I have read this submission. I believe that I have an appropriate level of expertise to confirm that it is of an acceptable scientific standard.

# Peer review: Referee response for version 1

**DOI:** 10.5256/f1000research.6684.r8300

**Published:** 2015-04-21

**Authors:** Dylan Wagner

**Affiliations:** 1 Department of Psychology, Ohio State University, Columbus, OH, USA

This data note describes a dataset consisting of time indices and annotations for the emotional content of scenes from the movie Forest Gump. The primary intent of this dataset is to encourage analyses of real-world cognition during the viewing of naturalistic stimuli and, as such, it is meant to accompany a previously released dataset of functional neuroimaging data using the same movie stimulus as used here.

I have only minor quibbles:

- The authors may wish to indicate which observers were familiar with the movie prior to annotation. 
- I recommend adding a section in which the authors discuss some of the potential issues that may limit the correspondence between the emotion annotation dataset and the neuroimaging dataset of the same movie. Specifically, the use of random scene order during annotation (but not during movie viewing) or the gender split among observers who annotated the audio movie and those that annotated the audiovisual movie. 
- At the expense of looking a gift horse in the mouth, an ipython notebook capable of generating all figures and descriptive data would have been marginally preferable to a script.

 Finally, I would like to note that the release of this dataset along with the associated neuroimaging data previously released by members of this group represents an extremely generous sharing of resources. I commend the effort and look forward to seeing what emerges from researchers looking into this treasure trove of data.

I have read this submission. I believe that I have an appropriate level of expertise to confirm that it is of an acceptable scientific standard.

# Response

**Published:** 2015-04-22

**Authors:** Michael Hanke

**Affiliations:** Otto-von-Guericke-Universität Magdeburg, Germany

Thanks a lot for the swift and positive feedback!

re familiarity: I think it is fair to say that by the time when observers started the actual annotation, all of them were somewhat familiar with the movie, due to the pre-tests we ran on the feasibility of particular annotation approaches -- independent of whether they saw the movie before. We'll add a corresponding statement.

re correspondence limitation: we could prepare a dedicated summary of aspects that may have an impact. However, other than the two aspects mentioned by the reviewer it may only be a question of "detection probability" which should be reflected in the inter-observer agreement. The issue of scene order randomization should emphasize the portrayed emotions as opposed to lower-frequency mood modulations induced by the movie (see procedure description). The gender split is an issue, but its impact is unclear. Most likely it causes the annotations to be less representative for the opposite gender. However, the annotation procedure encouraged a repeated, rather analytic dissection of the stimulus, in contrast to a single-pass introspection of how a scene "felt". This may compensate for an initial gender bias in the perception of the stimulus.

re ipython notebook: great suggestion! We'll convert the script into a notebook, and will provide both.

Thanks again!
